# The microbiota–proteostasis axis: implications in neurodegenerative diseases

**DOI:** 10.1098/rstb.2025.0265

**Published:** 2026-10-01

**Authors:** Daniel M. Czyż, Brittani M. Brown

**Affiliations:** ^1^ Department of Microbiology, Institute of Food and Agricultural Sciences, University of Florida, Gainesville FL, USA; ^2^ McKnight Brain Institute, University of Florida, Gainesville FL, USA; ^3^ Emerging Pathogens Institute, University of Florida, Gainesville FL, USA

**Keywords:** proteostasis, microbiota, gut–brain axis, microbiota–proteostasis axis, neurodegenerative diseases, Alzheimer's disease, Parkinson's disease, bacteria

## Abstract

Disruption of protein homoeostasis, or proteostasis, is a hallmark of protein conformational diseases (PCDs), including Alzheimer's and Parkinson's disease. These disorders are characterized by progressive protein aggregation and cellular dysfunction, yet no effective therapies exist. Emerging evidence indicates that microbial communities influence host proteostasis, giving rise to the concept of the microbiota–proteostasis axis. Microbes and their products can modulate host proteins by engaging with host proteostasis. As such, microbial dysbiosis has been linked to proteostasis disruption through the production of metabolites, extracellular vesicles, functional amyloids, and toxins or effector proteins capable of seeding or destabilizing host proteins associated with PCDs. These microbial factors have been shown in experimental models to converge on key proteostasis pathways, including protein synthesis, folding and clearance, thereby potentially reducing cellular buffering capacity and lowering the threshold for proteotoxic collapse. In parallel, microbiota-driven influence on inflammatory responses and immune signalling further amplifies systemic proteostasis disruption. In this review, we synthesize emerging evidence defining the microbiota–proteostasis axis and highlight how microbial factors influence host proteostasis. Importantly, these host–microbe interactions often precede neurodegeneration, suggesting potential for early detection and intervention. Together, these insights support targeting the microbiota as a potential strategy to enhance proteostasis and delay or prevent neurodegenerative disease.

This article is part of the Theo Murphy meeting issue ‘ProteostaSys: a systems view of proteostasis’.

## 1. Introduction

Neurodegenerative protein conformational diseases (PCDs), like Alzheimer's disease (AD), Parkinson's disease (PD) or amyotrophic lateral sclerosis (ALS), form a diverse group of progressively debilitating disorders characterized by misfolding and aggregation of host-encoded metastable proteins [1]. This well-established, cell-intrinsic mechanism is distinct from the contributions of exogenous microbial products—an often overlooked influence that we focus on in this review. Despite differences in clinical presentation, these complex diseases share several pathological features, as they manifest owing to malfunctioning or overwhelmed cellular protein quality-control mechanisms that evolved to ensure optimal protein synthesis, folding, transport and clearance, through orchestrated responses collectively known as the proteostasis network [2]. The onset of PCDs is commonly attributed to uncontrollable variables such as ageing [3], genetic predisposition [4] and environmental stressors [5]; however, their aetiology is far more complex and remains elusive. The proteostasis network functions to buffer the cellular toxicity associated with misfolded proteins induced by the aforementioned factors, and disruptions to this network can shift the system towards progressive, self-amplifying pathology, such as that observed across all neurodegenerative PCDs.

Studies on the pathogenesis of PCDs have traditionally focused on the central nervous system (CNS), where disease-associated proteins, such as amyloid-β, tau and α-synuclein, are widely implicated in neuronal toxicity. However, what if key disease-initiating or disease-modifying targets reside within the human body, not in the CNS, but rather at the periphery? Such a hypothesis has been postulated for PD and supported by emerging evidence in humans and murine models [6–8]. The concept of proteostasis was first introduced to describe the dynamic networks that buffer the functional proteome, with a primary focus on cell-intrinsic mechanisms but limited consideration of cell-extrinsic modulators that may influence proteostasis at the organismal level. Evidence from *in vitro* systems, animal models and human association studies increasingly indicates that proteostasis is influenced not only by cell-autonomous intracellular pathways but also by extrinsic systemic factors, including microbial metabolism, host inflammatory responses that are modulated by microbial communities and microbiota-derived products. Such an integrated view has broadened the classical approach to PCDs from a purely neuronal perspective to a more holistic, systems-level view. In particular, the human gut microbiota (HGM) has been increasingly implicated in the pathogenesis of PCDs [9]. The HGM broadly refers to the full complement of microorganisms inhabiting the human gastrointestinal tract, estimated to harbour 10^13^–10^14^ bacterial cells, encoding 100–150 times more genes than the human genome and capable of synthesizing a vast array of bioactive factors that profoundly influence host physiology [10,11]. For example, certain gut microbes produce short-chain fatty acids (SCFAs), including butyrate, propionate and acetate, through the fermentation of dietary fibre [12], which have been shown in multiple *in vitro* and animal models to improve the intestinal barrier, inhibit protein aggregation and reduce oxidative stress [13–16]. By contrast, other detrimental microbes are capable of generating amyloidogenic proteins or inflammatory signals that can promote protein misfolding and aggregation [17]. Understanding the balance between commensal and proteotoxic pathogenic microbes and their influence on host proteostasis may reveal microbial signatures that predispose to or protect against PCDs.

Dysbiosis of the HGM is characterized by decreased microbial diversity, the expansion of pathogenic taxa and the loss of protective commensal microbes [18]. These profound compositional changes, although often asymptomatic, are consistently associated with PCDs [9], supporting the hypothesis that microbial communities may influence host proteostasis. Notably, microbial diversity of the HGM declines with age [19], and maintaining a ‘healthy’ gut is emerging as a protective factor against age-associated disorders, including PCDs [20]. While experimental studies in model organisms, such as *Caenorhabditis elegans*, have demonstrated that specific microbial taxa can directly influence host proteostasis and the stability of disease-associated proteins [13,21,22], human studies have primarily identified correlations between microbial composition and PCD risk [23]. However, the molecular mechanisms underlying microbial-mediated proteotoxicity or protection remain poorly understood. In agreement with the role of the HGM on host proteostasis, emerging evidence suggests that other microbial populations outside the gut may also influence disease pathogenesis, probably through their role in modulating proteostasis components. This review examines how microbes and their products interface with host proteostasis through the emerging gut–proteostasis axis within the broader microbiota–proteostasis framework. We examine mechanistic links between HGM and neurodegenerative pathologies and propose that the HGM is a proteostasis-modifying target and a key contributor to the development of proteinopathies. With the emergence of this new modifiable target, the HGM offers a promising, yet underexplored, therapeutic avenue. Modulating microbial communities through dietary intervention, pre-, pro- and postbiotics, phage therapy, antibody-based therapeutics or faecal microbiota transplantation, among other approaches, could restore microbial eubiosis and enhance proteostasis, thereby providing accessible and potentially viable strategies to prevent or slow the progression of neurodegeneration.

## 2. Microbial modulation of host proteostasis

The pathology of neurodegenerative PCDs involves a cascade of molecular malfunctions initiated when proteins fail to achieve their native three-dimensional conformations [24]. Genetic mutations, oxidative stress, toxins and age-related decline in cellular functions [25], among other factors, can destabilize proteins and generate folding intermediates with exposed hydrophobic residues that seed aggregation by forming soluble oligomers and protofibrils [26]. Increasing evidence suggests that soluble oligomers may exert the most cytotoxicity owing to their ability to compromise membrane integrity, impair synaptic activity and propagate prion-like pathology [27,28]. Aberrant peptide assemblies can further transition into insoluble β-sheet-rich amyloid fibrils like the amyloid-β plaques observed in AD and α-synuclein-rich Lewy body inclusions associated with PD [1,29]. Amyloid assemblies can exhibit both toxic gain-of-function, resulting from new, harmful properties acquired upon misfolding and aggregation, and toxic loss-of-function, affecting the function of essential proteins and molecular chaperones, either directly or through sequestration [30,31], thus affecting the proteostasis threshold. The accumulation of misfolded proteins both intra- and extracellularly is a hallmark of PCDs, where intrinsically metastable proteins from within the proteome of the affected individual assemble into a spectrum of toxic species ranging from soluble oligomers to insoluble inclusions, including neurofibrillary tangles, Lewy bodies and amyloid plaques [1].

While diverse microbes, including bacteria, viruses, fungi and protozoa, can influence the stability of disease-associated proteins and contribute to their misfolding and aggregation through numerous direct and indirect mechanisms, this review focuses on bacteria. Notably, the broad diversity of microbes implicated in PCD pathology suggests that many exert their effects by modulating host proteostasis upstream of protein folding, rather than through direct interactions with individual aggregation-prone proteins. This concept is supported by numerous *in vitro* studies and animal models, in which bacteria and their products have been shown to alter cellular stress response pathways [32–34], deliver amyloidogenic or chaperone-sequestering proteins capable of cross-seeding host aggregates [35], inhibit components of the ubiquitin-proteasome system (UPS) [36,37], perturb protein synthesis [38] and trigger systemic inflammation [39], among other mechanisms (table 1). Direct interactions between microbial factors and host disease-associated proteins, such as those observed between *Porphyromonas gingivalis* and tau [75], also occur and are discussed in this review. Emerging evidence suggests that microbes probably affect host proteostasis in the prodromal stages of disease, emphasizing the potential early changes and markers that could predict disease pathogenesis. By altering cellular homoeostasis, inflammatory responses, metabolic balance or stress response capacity, microbes may reduce the cell's ability to buffer endogenous intrinsically metastable proteins, thereby facilitating their misfolding and aggregation and ultimately contributing to disease pathogenesis (figure 1).

**Table 1.** Representative microbial taxa and their bioactive products that modulate host proteostasis pathways. (The table highlights representative microbial taxa and bioactive products reported to influence host proteostasis pathways implicated in disease pathology. ‘*In vitro*’ refers to cell culture, tissue culture or test-tube-based studies performed outside an intact organism. To provide additional disease-relevant context, some references listed include review articles linking specific microbes to disease development or progression.)

genus/species	key bioactive product	pathway	model	reference
*Akkermansia muciniphila*	3-sucCA cross-feeding	symbiosis; barrier protection; mucin degradation	human, mouse	[40,41]
*Bacteroides fragilis*	fragilysin	barrier disruption; inflammation	*in vitro*, mouse	[42–44]
*Bacteroides uniformis*	3-sucCA	barrier protection; cross-feeding	human, mouse	[40,45]
*Bilophila wadsworthia*	H₂S	mitochondrial stress; inflammation	mouse	[46,47]
*Brucella* spp.	VceC, TcpB, Bsp	UPR activation; inflammation	*in vitro*, human	[32,48–51]
*Chlamydia trachomatis*	CT288, Tarp	UPR activation; inflammation	human, *in vitro*	[52,53]
*Coxiella burnetii*	CirB	UPS disruption	mouse, *in vitro*	[36]
*Desulfovibrio* spp.	H₂S	α-syn aggregation	*Caenorhabditis elegans*	[46,54]
*Escherichia coli*	CsgA/B, ElaD, CNF1 toxin, polyamine	cross-seeding (amyloid), UPS disruption, chaperone sequestration, mitochondrial stress	*C. elegans*, mouse, rat, *in vitro*	[13,17,55–61]
*Fusobacterium nucleatum*	LPS	aggregation; inflammation	mouse, *in vitro*	[62,63]
*Helicobacter pylori*	VacA, CagA, LPS	UPR activation; inflammation	human, mouse, *in vitro*	[64–67]
*Klebsiella* spp.	LPS	aggregation; inflammation; mitochondrial stress	*C. elegans*, mouse	[13,21,68]
*Legionella pneumophila*	Lgt1–3	UPR inhibition; translation inhibition	*in vitro*	[69,70]
*Listeria monocytogenes*	LLO	ER stress; mitochondrial dysfunction	*in vitro*	[71,72]
*Mycobacterium* spp.	ESAT-6, HBHA	ER stress	*in vitro*	[73,74]
*Porphyromonas gingivalis*	gingipains (Rgp/Kgp), DNA, LPS	tau cleavage; cross-seeding (DNA); inflammation; translocation through BBB	human, mouse, rat, *in vitro*	[75–79]
*Prevotella corporis*	possible metabolite	aggregation; HSR	*C. elegans*	[34]
*Proteus mirabilis*	HpmA haemolysin, LPS	aggregation; α-syn interaction; autophagy (mTOR); ENS propagation; inflammation	*C. elegans*, mouse	[13,80]
*Pseudomonas aeruginosa*	FapC, exotoxin A	aggregation; cross-seeding (amyloid); translation inhibition; inflammation	*C. elegans*, *Danio rerio*, *in vitro*	[13,81–84]
*Salmonella enterica*	curli, SopA, SseL, SlrP, TMA	aggregation; cross-seeding (amyloid); UPS disruption, UPR–UPS crosstalk; metabolic stress; inflammation	*C. elegans*, *in vitro*, mouse	[13,85–88]
*Shigella* spp.	Shiga toxin	aggregation; UPR activation	*C. elegans*, *in vitro*	[13,89]
*Streptococcus mutans*	amyloids, imidazole propionate	cross-seeding (potential)	*in vitro*, human, mouse	[90–92]
*Treponema denticola*	dentilisin protease	cytoskeleton disruption; tau pathology	mouse, *in vitro*, human	[93–98]
*Vibrio cholerae*	cholera toxin	ER stress	*in vitro*	[99,100]

**Figure 1.**
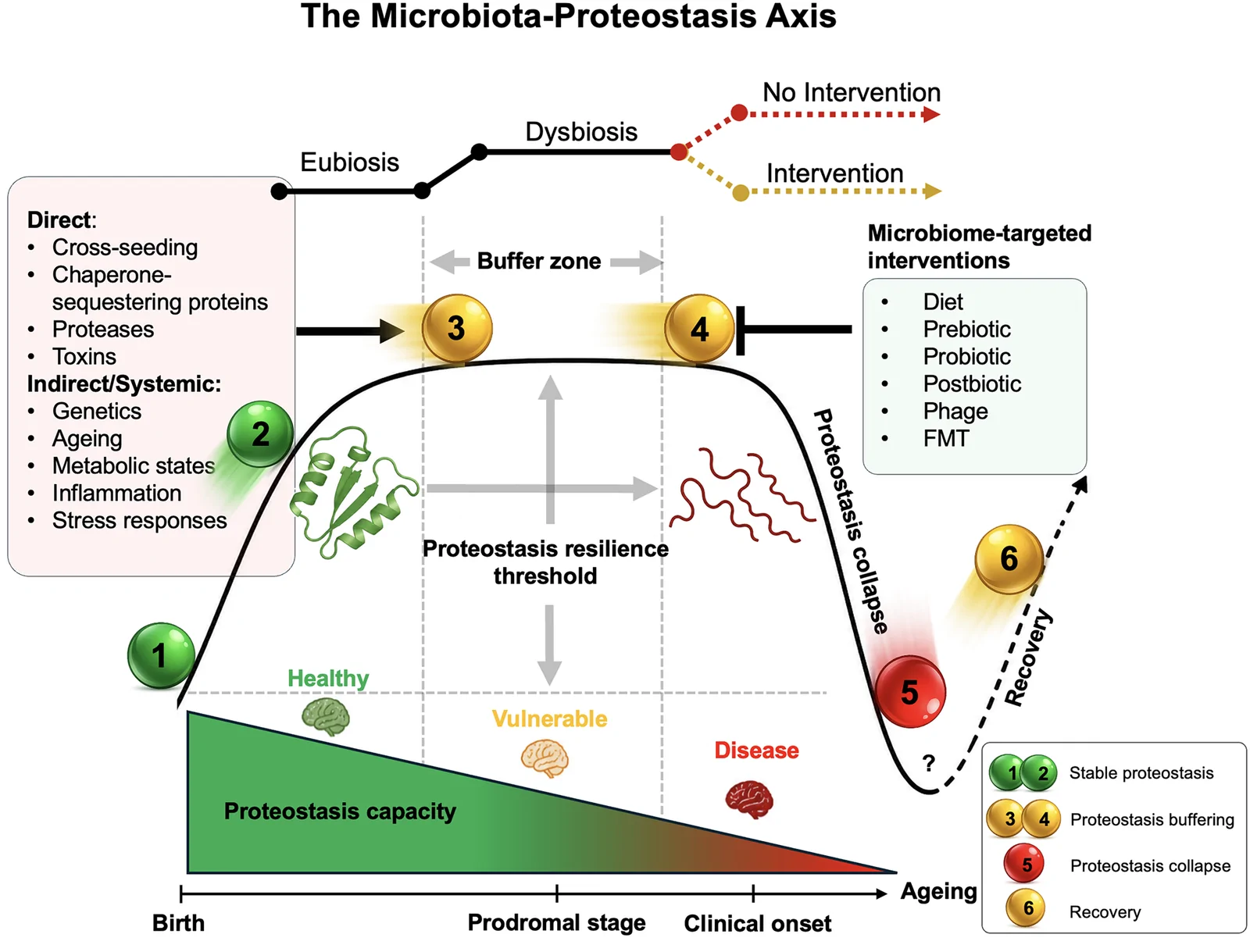
The microbiota–proteostasis axis. Conceptual model illustrating how microbiota-derived factors and gut-targeted interventions influence host proteostasis resilience across ageing and disease progression. Under eubiotic conditions, the host proteostasis network maintains protein folding homoeostasis and buffering capacity despite proteotoxic insults. Indirect/systemic factors, including genetics, ageing, metabolic dysfunction, inflammation and host stress responses, together with direct microbial mechanisms such as cross-seeding, chaperone-sequestering proteins, proteases and toxins, progressively disrupt proteostasis resilience. Within the ‘buffer zone,’ compensatory mechanisms within the proteostasis network preserve proteome stability despite accumulating perturbations. However, once the proteostasis resilience threshold is exceeded, buffering capacity collapses, accelerating protein misfolding, aggregation and disease onset. The declining proteostasis capacity gradient illustrates the age-associated reduction in host resilience over time and chronic insults, progressing from prodromal stages to clinical disease. Dysbiosis contributes to proteostasis collapse through both direct and indirect/systemic mechanisms, whereas microbiome-targeted interventions, including dietary modulation prebiotics, probiotics, postbiotics, bacteriophage (phage)-mediated strategies and faecal microbiota transplantation (FMT), may extend the buffering capacity, help restore proteostasis resilience and support recovery. Numbered spheres indicate progressive functional states: stable proteostasis (1 and 2), proteostasis buffering (3 and 4), proteostasis collapse (5) and recovery (6). The figure was created in Microsoft PowerPoint using original graphical elements and OpenAI-generated visual components (spheres, protein and brains).

An estimated 90–95% of PCDs, such as AD or PD, are sporadic, with disease onset and progression influenced by a complex interplay of exogenous and endogenous factors, including genetics, ageing, environmental exposures and the microbiome [101,102]. Inter-individual variability in microbial composition probably contributes to the heterogeneity in the prodromal period and disease susceptibility. In addition, differences in the capacity to mount protective stress responses following environmental or physiological insults may further influence disease progression, even in genetically predisposed individuals. For example, a recent study suggests that chronic exposure to elevated temperature enhanced proteostasis through the activation of protective stress responses, particularly the heat shock response (HSR) [103]. Genetic variants associated with increased disease susceptibility are well studied, but their mechanisms remain elusive. For instance, apolipoprotein E4, one of the strongest genetic risk factors for late-onset AD [104,105], was recently shown through proteomic profiling to induce conserved pro-inflammatory immune signatures enriched for pathways associated with host responses observed during various infections, including pathogenic *Escherichia coli* [106]. These results suggest that genetically driven immune remodelling may alter an individual's susceptibility to chronic inflammatory and proteotoxic stressors, ultimately affecting the organismal ability to buffer misfolded proteins (figure 1).

## 3. Microbiota–proteostasis axis: a systems-level framework

Emerging evidence suggests that microbial influence extends beyond classical gut–brain communication and instead reflects a broader capacity to regulate host proteostasis at the organismal level. Rather than acting solely through neural, immune, endocrine or metabolic signalling routes, microbes and their products can directly interact with components of the cellular proteostasis machinery, thereby influencing the host's threshold capacity to buffer a stable and functional proteome (figure 1). This broader perspective gives rise to the concept of a microbiota–proteostasis axis: a systems-level regulatory pathway through which microbial metabolites, amyloids, toxins, effectors and other microbe-derived cues influence protein folding, stability, localization and clearance across tissues. Importantly, this axis is not restricted to the gastrointestinal tract. While the gut–proteostasis axis represents a major and most well-characterized instantiation of this pathway, analogous mechanisms are likely to operate across additional microbial niches, including those colonizing the oral cavity, respiratory tract, vagina, skin, individual organs and even the eye [52], as recently supported by human studies identifying associations between ocular microbial colonization and AD.

In contrast to the gut–brain axis, which emphasizes bidirectional flow of information between the gut and the CNS, the microbiota–proteostasis axis centres on modulation of host protein quality control itself, both centrally and peripherally. This view is particularly important given that PCDs exhibit substantial peripheral pathology and systemic proteostasis disruption that extends beyond the CNS and, in some cases, may precede neurodegeneration and clinical symptoms [107]. Therefore, understanding how microbiota modulate organism-wide proteostasis will be essential for defining microbial contribution to PCD onset and progression.

## 4. Routes of microbial signal transmission to the central nervous system

The gut–brain axis is defined by a bidirectional communication network through which gut microbes influence the CNS via neural, immune, endocrine and metabolic pathways. These interactions occur through key regulatory interfaces, including the vagus nerve, systemic circulation and the blood–brain barrier (BBB) [108,109]. This framework positions the gut microbiome as an active regulator of neuronal and glial physiology, capable of indirectly affecting proteostasis within the CNS. At the same time, it provides the anatomical and molecular infrastructure required for the trafficking of microbial products and signals along the gut–proteostasis axis.

### (a) Neural pathways

One of the most direct conduits of gut–brain communication is the enteric nervous system (ENS) and vagal afferent fibres. Gut microbes modulate vagal signalling through numerous mediators, including SCFAs [110], tryptophan metabolites [111] and neuroactive compounds such as γ-aminobutyric acid (GABA) and serotonin precursors [112,113]. Studies in mice reveal that enteroendocrine cells sense these microbial signals and modulate vagal afferent firing accordingly [114,115]. Vagal afferents relay information via the nodose ganglion to the nucleus tractus solitarius and subsequently engage ascending brainstem circuits, conveying peripheral signals to higher-order brain regions [114,116,117]. The ENS locally integrates microbial and immune signals via enteric glia and cytokines to regulate gut motility, secretion and cross-communication with the central autonomic network [118–120]. Animal studies demonstrate that vagotomy blunts some behavioural effects of microbiome perturbations, thereby supporting the relevance of neural routes in microbiota-mediated brain effects [112,121]. Epidemiological studies in Danish and Swedish populations have also suggested that truncal vagotomy is associated with reduced risk for PD, although the strength and consistency of this association remain debatable [122,123].

### (b) Immune pathways

Another major route within the gut–brain axis is through immune and inflammatory mediators. The gut microbiome exerts influence over baseline intestinal and systemic immunity by shaping immune responses at the mucosal interface, where microbial products and controlled immune responses across the epithelial barrier influence lamina propria immune cells. Under conditions of dysbiosis or ageing, barrier dysfunction may permit increased translocation of microbial products into the systemic circulation [124,125]. Microbial-associated molecular patterns, such as lipopolysaccharides (LPS), peptidoglycans and bacterial extracellular vesicles (EVs), are all capable of triggering innate immune receptors on epithelial, dendritic and myeloid cells, leading to cytokine production [126]. These molecules exert enhanced influence under increased gut permeability. Cytokines and chemokines can cross or signal across the BBB to influence microglial activation, astrocyte states and neuronal stress responses [127], thereby propagating systemic inflammation into the CNS, potentially contributing to the pathogenesis of PCDs [39,128]. Dysbiosis-associated inflammation can further increase BBB permeability, enhancing CNS exposure to immune mediators, microbial products and even microbes [68,129]. By contrast, microbial metabolites, including SCFAs and tryptophan-derived indoles, primarily signal through G-protein-coupled receptors and nuclear receptors such as the aryl hydrocarbon receptor to promote anti-inflammatory effects and immune homoeostasis [130,131]. As such, a decrease in taxa producing such mediators, particularly butyrate, is often associated with PCDs and other neurological conditions [132,133]. Recent evidence indicates that muscularis macrophages that specialize in ENS and intestinal homoeostasis also play a role in the dissemination of α-synuclein pathology from the intestine into the CNS [134].

### (c) Endocrine pathways

The endocrine system is another major component of gut–brain communication, with the gut microbiota increasingly recognized as functioning similarly to an endocrine organ, producing a diverse array of signalling molecules with cross-tissue activities. The gut microbiome also directly modulates neuroendocrine circuits via the hypothalamic–pituitary–adrenal (HPA) axis. Microbial metabolites influence enteroendocrine cells to alter gut hormone levels such as GLP-1, PYY and ghrelin [117], which regulate appetite, stress responses and central neurophysiology. Microbial modulation of peripheral serotonin production—approximately 90% of which is synthesized in the gut—also shapes CNS signalling [135]. Activation or dysregulation of the HPA axis leads to cortisol release, which, in turn, acts on central neurons and glia to modulate stress responses, inflammation and protein quality-control pathways within the proteostasis network [136,137].

### (d) Metabolic pathways

Beyond neural, immune and endocrine signalling, microbial metabolism constitutes a distinctive signalling route that facilitates gut–brain communication. With over 2000 unique bacterial species [138], the human gut functions as a reservoir of microbial bioactive metabolites, including SCFAs, bile acid derivatives, polyamines and indole derivatives, which can enter systemic circulation and influence host cellular metabolism, redox balance and proteostasis [139,140]. These metabolites can cross or signal across the BBB, modulate host mitochondrial function, regulate histone acetylation and gene expression and influence the activity of molecular chaperones, autophagy and protein quality-control pathways [140]. Through these mechanisms, microbial metabolism exerts a continuous and systemic influence on neuronal vulnerability to protein misfolding and aggregation, thereby providing a mechanistic link between microbial activity and PCD pathogenesis independent of classical neural, immune or endocrine signalling.

### (e) Physical route

Emerging evidence indicates that, in addition to transmitting microbial and metabolic signals, vagal and enteric circuits may provide a direct anatomical route for the propagation of disease-associated proteins [8,141–143]. Experimental models support the Braak hypothesis, which suggests that misfolded α-synuclein can originate in the gut and ascend to the brainstem via trans-synaptic propagation along the vagus nerve [144]. Murine studies have demonstrated that inoculation of α-synuclein fibrils or overexpression constructs into the intestinal wall or ENS leads to the sequential appearance of α-syn pathology in the dorsal motor nucleus of the vagus, locus coeruleus and midbrain, a process that is attenuated or abolished by vagotomy [7,8,145]. Although aspects of this model remain debatable in human studies as described in the Danish and Swedish populations mentioned above, animal models consistently demonstrate gut-to-brain propagation under defined conditions. Similar studies indicate that tau and amyloid-β assemblies introduced into the gut can travel to connected brain regions and seed aggregation in a prion-like manner [141,146]. These findings suggest that ENS–vagus communication could serve not only as a conduit for microbial signalling but also as a highway for the spread of proteopathic seeds from the periphery to the CNS, perhaps even those of microbial origin.

Together, these neural, immune, endocrine, metabolic pathways and physical vagus nerve routes illustrate the diverse ways in which gut microbiome signals could potentially access the CNS in humans. Yet, despite the breadth of these communication pathways, they explain only *how* microbial signals and proteotoxic species can reach the brain, rather than *how* these signals ultimately shape host proteostasis by targeting the stability, folding and clearance of host proteins. Increasing evidence suggests that microbial metabolites, structural components and secreted effectors may directly modulate the host proteostasis machinery, influencing protein stability, folding and clearance and ultimately increasing vulnerability to aggregation. This perspective extends the traditional gut–brain paradigm towards a broader conceptual framework of the gut–proteostasis axis, in which microbial signals may systemically influence the host proteome across both central and peripheral tissues.

## 5. Gut–proteostasis axis

Gut dysbiosis in humans and animal models is consistently associated with PCDs, including PD, AD and ALS [147–149], supporting the hypothesis that microbes influence host proteostasis, ultimately modifying disease pathogenesis. In germ-free mice colonized with faecal microbiota from PD patients, motor deficits and neuroinflammation are exacerbated relative to colonization with microbiota from healthy donors [147], implicating microbial composition as a functional modifier of disease severity. Isolates of *Desulfovibrio* collected from PD patients similarly enhance α-synuclein aggregation and reduce survival in *C. elegans* [54]. These findings suggest that microbial metabolites, secreted effectors or other components may influence the host proteostasis network [149]. An increasing number of studies have identified microbial metabolites as critical mediators of gut–proteostasis signalling [148,149]. For example, SCFAs regulate chromatin accessibility, modulate expression of stress response genes and strengthen the intestinal and BBB integrity [130]. Within the context of proteostasis, butyrate has been found to influence autophagy, thereby enhancing the clearance of misfolded proteins. In human studies, metagenomic surveys consistently reveal the depletion of SCFA-producing taxa and enrichment of opportunistic pathogens in PD cohorts [150,151]. Moreover, perturbations in SCFA levels caused by dysbiosis have been linked to altered proteomic signatures in the CNS, including shifts in metabolic enzymes, chaperone networks and stress response proteins [127,152]. Such signatures may represent an early event in disease pathogenesis, offering potential biomarkers for predisposition or the detection of prodromal stages of neurodegenerative diseases.

### (a) Direct mechanisms: microbial influence on host proteostasis

#### (i) Cross-seeding agents and chaperone sequestration

Representative examples of bacterial taxa, their bioactive products and the host proteostasis pathways they target are summarized in table 1. The gut microbiome often harbours proteotoxic bacteria that are associated with PCDs. Many of these detrimental species, while associated with enteropathogenic infections, colonize the gut asymptomatically [153–157]. One of the major families of such enteropathogenic bacteria is *Enterobacteriaceae*, which is known for its production of functional amyloids—structural biofilm proteins that resemble host amyloidogenic sequences known to cross-seed with disease-associated proteins and accelerate their aggregation [9,17,158]. The curli system of *E. coli* exemplifies this phenomenon. Curli subunits CsgA and CsgB co-localize with α-synuclein, where they accelerate aggregation and exacerbate motor impairment in experimental models [55]. Similar cross-seeding extends to amyloid-β, SOD1 and huntingtin, suggesting a broad capacity of microbial amyloids to amplify host misfolding [55–57]. *Pseudomonas aeruginosa* is a ubiquitous and opportunistic pathogen of skin and respiratory infections but also commonly found in the gut of healthy individuals [156,159]. These bacteria secrete the FapC amyloid, whose β-solenoid architecture provides structural complementarity for Aβ nucleation [81,82]. Additionally, *P. aeruginosa* secretes protein aggregates whose proteotoxic effects are amplified when butyrate levels are reduced [22]. *Salmonella enterica*, another curli-producing organism, synthesizes amyloid fibres that are structurally conserved across *Enterobacteriaceae*. While direct evidence of cross-seeding by *Salmonella* curli is limited, curli fibres from related species, such as *E. coli*, have been shown to accelerate the aggregation of α-synuclein and amyloid-β and to accelerate neurodegeneration in murine model systems [57,58], suggesting a shared proteotoxic potential. Gut-derived bacterial amyloids have been implicated in proteotoxicity across several models [59]. The oral microbiota harbours amyloid producers. For example, *Streptococcus mutans* is known for its ability to produce functional amyloids [90,91], including amyloid-forming adhesins and biofilm-associated proteins that contribute to extracellular matrix stability and persistence within the oral niche. These amyloid structures share biophysical features with classical cross-β amyloids, suggesting their potential to interact with host amyloidogenic proteins and influence their aggregation. The presence of such amyloid proteins within any of the four aforementioned routes could sequester chaperones away from the endogenous proteome, ultimately decreasing the cellular capacity to buffer protein folding (figure 1). The bacterial amyloids could mimic destabilized host proteins and directly interact with disease-associated proteins. In addition to microbial amyloids, bacterial nucleic acids may also promote tau aggregation. For example, *P. gingivalis* DNA has been shown to catalyse tau aggregation *in vitro*, an effect abolished by DNase treatment [76].

In parallel, microbial amyloids and misfolded proteins probably sequester molecular chaperones [160], ultimately supporting the relevance of the microbiota–proteostasis axis in disease pathology. Sequestration of HSPs by destabilized host proteins has been observed across families of molecular chaperones [161]. Other sequestering mechanisms could also include chaperones that prevent aggregation (holdases) [162], those that promote aggregation to alleviate soluble prion toxicity [163] and those that engage in disaggregation [164]. Our studies employing *C. elegans* further suggest that bacteria-derived protein aggregates and changes in the microbial proteostasis can impair host proteostasis, leading to the disruption of the folding environment [22]. Together, these findings support a model in which microbial amyloids may destabilize host proteostasis both by directly accelerating misfolding through cross-seeding and by depleting molecular chaperone reserves, thereby lowering the threshold for proteotoxic collapse (figure 1).

#### (ii) Proteolytic destabilization of host proteins

Bacterial pathogens can destabilize host proteostasis through the secretion of extracellular proteases that modify the host proteome [165]. These enzymes target a broad range of host substrates, including mucins, tight-junction proteins, cytokines, immunoglobulins and components of the ubiquitin-proteasome system, thereby disrupting protein homoeostasis and host cellular integrity. Among oral bacterial taxa, a keystone pathogen in periodontitis, *P. gingivalis,* represents a canonical example of a bacterium that exhibits proteotoxic effects linked to Alzheimer's pathology [77]. This bacterium secretes gingipains—potent Rgp and Kgp cysteine proteases—that cleave tau, destabilize microtubules and accelerate tau hyperphosphorylation, a key step that drives neurofibrillary tangle formation [75]. Chronic exposure to gingipains and LPS activates microglia and inflammatory signalling pathways such as p38 MAPK and JNK, exacerbating tau pathology and Aβ deposition [78,79,166]. Consistent with these mechanisms, post-mortem analyses of human brains have detected *P. gingivalis* DNA, gingipain antigens and elevated inflammatory mediators in Alzheimer's patients [75]. Importantly, in mouse models of oral infection, pharmacological inhibition of gingipains reduces brain bacterial load, Aβ accumulation and neuroinflammation, supporting a causal role for this pathogen in disrupting neuronal proteostasis [75]. Beyond *P. gingivalis*, several other oral anaerobes have been associated with similar neurotropic and proteotoxic effects. For example, infection with *Treponema denticola* has been shown in a mouse model to promote hippocampal Aβ deposition and neuronal apoptosis [93]. This bacterium has been frequently found in the brains of AD [94,95]. It produces dentilisin, a surface protease implicated in neuronal actin cytoskeleton perturbation [96], epithelial tight-junction disruption [97] and induction of tau pathology [98]. Dentilisin has been detected in *T. denticola* EVs [167], raising the possibility of BBB permeability and probable barrier disruption [168]. Another emerging oral and gut pathobiont associated with exacerbation of AD pathology is *Fusobacterium nucleatum* which enhances microglial activation and tau phosphorylation in transgenic AD murine models [62]. EVs produced by *F. nucleatum* are also known for their pro-inflammatory properties [63]. Extending beyond oral-associated taxa, gut-resident bacteria also contribute to proteostasis disruption through analogous protease-mediated mechanisms that impact barrier integrity and systemic inflammation. *Bacteroides fragilis*, a gut-resident bacterium, secretes fragilysin, a zinc-dependent metalloprotease that cleaves E-cadherin, ultimately increasing gut permeability and triggering systemic inflammation, and is associated with AD [42–44]. Collectively, these observations suggest that proteases derived from chronic dysbiosis function as a systemic inflammatory reservoir capable of propagating proteotoxic stress along the microbiota–proteostasis axis.

#### (iii) Microbial toxin–mediated disruption of host proteostasis

Microbial toxins can play an essential role in remodelling host proteostasis by disrupting stress responses, protein degradation and trafficking pathways. Numerous bacterial pathogens, particularly intracellular species, produce toxins or effector proteins that target core components of the proteostasis network and can exert both local and systemic effects, ultimately contributing to protein misfolding and aggregation associated with PCDs.

Microbial toxins frequently target the unfolded protein response (UPR), a central regulator of endoplasmic reticulum (ER) proteostasis, to remodel the host folding environment and promote pathogen survival. For example, *Brucella* species secrete the effector VceC, which binds the ER chaperone BiP to induce UPR signalling and remodel ER architecture [48]. Additional effectors, including TcpB and Bsp proteins, further promote ER stress responses through disruption of the host secretory pathway [32,49]. Because many of these virulence factors are secreted or released extracellularly, their effects are not necessarily confined to the site of infection and may influence host proteostasis in distal tissues. Interestingly, neurobrucellosis, a rare CNS manifestation of *Brucella* infection, presents with PD-like symptoms [50,51], highlighting the potential neurological consequences of pathogen-mediated disruption of host proteostasis. Other intracellular pathogens selectively modulate specific branches of the UPR. For instance, *Legionella pneumophila* glycosyltransferase effectors Lgt1-3 inhibit XBP1 splicing and translation elongation [140,141], whereas *Chlamydia trachomatis* effectors, such as CT288 and Tarp, activate the IRE1 pathway through oligomerization of the receptor [53]. Bacteria can also induce UPR through calcium- and oxidative-stress signalling, a mechanism that is mediated by *Mycobacterium* effectors ESAT-6 and HBHA and the pore-forming toxin listeriolysin O (LLO) from *Listeria monocytogenes* [71,73,74]. In addition to inducing ER stress, *Listeria* LLO also disrupts mitochondrial function, further coupling bacterial toxin exposure to bioenergetic stress and proteostasis disruption [72].

Several enteric-origin toxins also converge on the ER chaperone BiP, a central regulator of protein folding and UPR signalling. For example, cholera toxin from *Vibrio cholerae* and Shiga toxin from *Shigella* interact with BiP [89,99], whereas the subtilase cytotoxin SubAB cleaves BiP, thereby triggering ER stress [169]. Similarly, the gastric pathogen *Helicobacter pylori* secretes vacuolating cytotoxin A (VacA), which activates the PERK branch of the UPR, while other virulence factors, such as cytotoxin-associated gene A (CagA) and LPS, can promote systemic and neuroinflammatory responses that disrupt proteostasis along the gut–brain axis, though probably via indirect mechanisms [64]. Indeed, *H. pylori* infection has been associated with several PCDs [65,66,170]. Interestingly, the effector protein CagA has also been reported to exhibit anti-amyloid properties and can be detected in EVs [67,171], suggesting that microbial proteins may influence amyloidogenic processes in distal tissues.

In parallel with UPR modulation, bacterial pathogens directly manipulate the UPS, a core component of protein quality control responsible for the recognition and degradation of misfolded proteins. The *S. enterica* effector protein SlrP, an E3 ubiquitin ligase, targets the ER-resident Hsp40 chaperone ERdj3 [85], linking UPS signalling to UPR activation and proteostasis disruption [85]. Another *Salmonella* effector, the SopA ubiquitin ligase, targets host TRIM E3 ligases, including TRIM56 and TRIM65, for ubiquitination and degradation, leading to the suppression of innate immune signalling and disruption of ubiquitin-dependent regulatory networks [86]. Given the roles of TRIM proteins in coordinating selective autophagy and stress signalling [172], their loss may impair protein quality-control pathways and reduce the cellular capacity to clear misfolded proteins, ultimately contributing to proteostasis imbalance. Additional *Salmonella* effectors further expand this disruption. SseL, a deubiquitinase (DUB), interferes with UPS-dependent protein degradation [87], while SteC facilitates cytoskeletal rearrangements that enhance *Salmonella*-mediated gut permeability [88]. In addition to well-characterized *Salmonella* factors, other bacterial pathogens also deploy DUBs to directly manipulate host protein quality-control pathways. For example, ElaD, an *E. coli*–expressed DUB present in the majority of isolates [60], targets host K63-linked ubiquitin chains, which are critical for autophagy and stress signalling, thereby impairing the clearance of misfolded proteins and altering proteostasis. Complementing this mechanism, uropathogenic *E. coli* secretes CNF1, a toxin that alters Rho GTPase signalling and disrupts UPS pathways, further compromising protein clearance [161].

Beyond ER- and UPS-associated mechanisms, bacterial toxins disrupt host proteostasis through additional pathways, including protein synthesis, mitochondrial dysfunction and EV-mediated dissemination. One of the most notable host proteostasis-modifying toxins is exotoxin A, produced by *P. aeruginosa*, an ADP-ribosyltransferase that modifies the diphthamide residue on eukaryotic elongation factor 2, thereby irreversibly halting protein synthesis [83]. This translational arrest prevents replenishment of short-lived regulatory and quality-control proteins, including molecular chaperones and proteostasis regulators, leading to progressive collapse of the cellular proteostasis network and accumulation of damaged proteins. As a result, cells experience severe proteostasis stress driven by an impaired protein synthesis and degradation imbalance. Notably, *P. aeruginosa* colonization has been linked to severe inflammatory responses and proteostasis disruption [13,84], processes that contribute to neurodegenerative pathology [173]. Another notable example of a gut microbial resident is the HpmA haemolysin from *Proteus mirabilis*, which induces α-synuclein aggregation in enteric neurons, with pathology propagating along the gut–brain axis in mice [80]. Colonization of mice with *P. mirabilis* induces α-synuclein aggregation in the enteric nervous system, with pathology ascending to the brainstem and substantia nigra through the vagus nerve, consistent with Braak's hypothesis [80]. Infected animals display dopaminergic neuron loss and motor dysfunction, which can be ameliorated by antibiotics, supporting a mechanistic role of *P. mirabilis* in disease progression through several mechanisms, including direct interaction of its effector, HpmA, with α-synuclein and its dysregulation of mTOR-mediated autophagy [80].

Importantly, many bacterial toxins and effectors can be packaged into EVs, which provide an efficient mechanism for systemic dissemination of microbial factors. For example, *Brucella* VceC and Bsp proteins [174], as well as VacA [175], Shiga-like toxins [176], *Listeria* LLO [177] and cholera toxin, have been detected in EVs [178]. These vesicles may enable microbial factors to influence host proteostasis at distant sites along the microbiota–proteostasis axis. Indeed, many of these bacteria have been associated with PCD pathogenesis or disease progression [179].

Together, these mechanisms demonstrate that microbial toxins converge on core proteostasis nodes, including the UPR, UPS, autophagy, mitochondrial function and translational control, thereby reducing the capacity of host cells to maintain protein homoeostasis and lowering the threshold for proteotoxic collateral damage in vulnerable tissue (figure 1).

### (b) Indirect systemic mechanisms: microbial influence on host proteostasis

#### (i) Bacterial metabolites

Bacteria produce a wide range of bioactive metabolites capable of interacting with host pathways, thereby affecting proteostasis, protein stability and, ultimately, disease pathogenesis. Among some of the best examples of bacterial metabolites that influence host proteostasis are SCFAs, secondary bile acids, polyamines and sulphur metabolites. SCFAs are extensively studied microbial metabolites implicated in neurodegenerative diseases [180,181]; particularly butyrogenic bacteria are depleted in PD patients’ gut metagenomes [182,183]. Butyrate supplementation has been shown to improve memory in AD mice, ameliorate pathology in the ALS murine model and directly suppress protein misfolding in tissue culture [184,185]. The abundance of Firmicutes, a bacterial phylum known for its butyrogenic properties, is significantly lower in animal models and the guts of patients with PCDs [148,186–188]. SCFAs, in particular butyrate, directly regulate chromatin accessibility, modulate expression of stress response genes and strengthen intestinal and BBB integrity [130]. Mechanistically, butyrate functions as a histone deacetylase inhibitor, promoting transcription of genes involved in stress responses, protein folding and protein quality-control pathways [189–191]. Through this epigenetic regulation, butyrate enhances expression of molecular chaperones and components of the proteostasis network [192]. Its aggregate-suppressive activity was previously shown to require SKN-1/Nrf2 and DAF-16/FOXO transcription factors [13]. Within the context of proteostasis, butyrate has been shown to influence autophagy, thereby enhancing the clearance of misfolded proteins [193,194]. In addition, butyrate supports mitochondrial function, protects against LPS-induced damage and reduces oxidative stress, processes that further enhance host proteostasis [195–197]. Human and animal model metagenomic analyses consistently identify associations between PCD pathogenesis and depletion of SCFA-producing taxa [150,198]. Moreover, perturbations in SCFA levels caused by dysbiosis have been connected to altered proteomic signatures in the CNS, including shifts in metabolic enzymes, chaperone networks and stress response proteins [199]. Butyrate serves as the primary energy source for colonocytes and plays a central role in maintaining intestinal barrier integrity—a function that decreases with age and is further exacerbated by the presence of enteropathogenic bacteria [200,201]. In line with the protective role of butyrogenic bacteria, *Prevotella corporis* was recently reported to activate a protective HSR in the absence of any exogenous stress, leading to increased expression of molecular chaperones and suppression of aggregation across multiple disease-associated proteins, including Aβ, α-synuclein, tau and polyglutamine expansions [34].

Secondary bile acids are microbial metabolic products generated through bacterial modification of primary bile acids, including cholic and chenodeoxycholic acids. Gut microbes convert these host-derived molecules through dihydroxylation, oxidation and epimerization reactions, producing secondary bile acids such as deoxycholic acid (DCA) and lithocholic acid (LCA) [202]. While bile acids serve important physiological roles in lipid digestion and metabolic signalling, secondary bile acids can exhibit cytotoxic and pro-inflammatory effects in both peripheral and CNS tissues [203]. Both DCA and LCA can disrupt BBB tight junctions, leading to increased permeability and enhanced exposure to circulating inflammatory and microbial products [204]. Hydrophobic bile acids also perturb cellular stress pathways and mitochondrial redox balance, creating a permissive environment for protein misfolding and impaired clearance, although direct effects on neuronal proteostasis remain to be fully defined [205]. Secondary bile acids can also influence the growth of specific gut microbial populations [206]. For example, *Bacteroides uniformis*, the abundance of which is higher in healthy controls compared to AD patients [45], expresses a β-lactamase-like enzyme that converts host-derived cholic acid into 3-succinylated cholic acid (3-sucCA), a lumen-restricted bile that selectively promotes the growth of *Akkermansia muciniphila* and alleviates metabolic dysfunction-associated steatohepatitis in mice by strengthening the mucus barrier [40]. Given that *A. muciniphila* upregulates tight-junction proteins and improves gut barrier integrity in multiple models, 3-sucCA-driven expansion of this taxon may similarly reduce gut permeability and systemic inflammation, with potential relevance to PCDs [41].

Polyamines such as spermidine and spermine represent another class of microbiota-derived metabolites that influence host metabolism, immune signalling and proteostasis [207–209]. These molecules regulate autophagy, modulate molecular chaperone expression and control translation. Multiple studies support the beneficial role of polyamines in maintaining proteostasis. For example, spermine has recently been shown to modulate the condensation behaviour of amyloidogenic proteins, including tau and α-synuclein, promoting autophagic clearance and mitigating neurodegenerative phenotypes in experimental models [210]. Consistent with this role in maintaining proteostasis, disruption of polyamine metabolism has been linked to muscle dysfunction and disease progression in ALS mouse models [211]. Additionally, polyamines are known for their role in regulating intestinal epithelial HSR [212]. Microbial polyamines can also exhibit detrimental effects on the host. For example, accumulation of a toxic intermediate of the spermidine synthesis pathway from *E. coli* or *Bacillus subtilis* results in mitochondrial dysfunction and activation of the mitochondrial UPR [61]. In agreement with these results, the differential effects of various polyamine intermediates on the stability of α-synuclein have also been observed [213].

Beyond polyamines, additional microbiota-derived metabolites influence host processes along the microbiota–proteostasis axis. Hydrogen sulphide (H_2_S) is a lipophilic metabolite and signalling molecule that readily crosses biological membranes, including the BBB. While high concentrations of H_2_S are produced by gut bacteria, host-derived H_2_S is generated enzymatically across tissues at much lower, tightly regulated levels [214]. The steep concentration gradient between microbial-derived H_2_S in the colon and host-regulated intracellular H_2_S may represent a critical threshold determinant of whether H_2_S signalling remains cytoprotective or becomes proteotoxic. In contrast to microbial H_2_S, which is often associated with cytotoxic effects, host-derived H_2_S has largely been described as protective [215]. H_2_S, therefore, remains a double-edged sword, probably reflecting its distinct sources and concentration-dependent effects, as studies have also shown its potentially protective role [216]. Excess microbial H_2_S can disrupt proteostasis through multiple mechanisms, including direct damage to mitochondrial proteins and inhibition of the respiratory chain [217]. Additionally, H_2_S depletes glutathione, weakening antioxidant defences and promoting oxidative stress that favours aggregation of amyloidogenic proteins such as α-synuclein [46]. *Bilophila wadsworthia* is an example of a gut pathobiont enriched under high-fat intake, often associated with a ketogenic diet [218]. Its expansion has been associated with gut inflammation and accelerated cognitive impairment in mouse models [47]. More broadly, several H_2_S-producing genera, including *Helicobacter*, *Treponema*, *Porphyromonas*, *Desulfovibrio*, *Salmonella*, *Klebsiella*, *Escherichia* and *Fusobacterium*, have been associated with PCDs and have been implicated in disruption of host protein folding and proteostasis pathways [13,21,46,54,66,68,75,96,170,219,220], suggesting that elevated microbial H_2_S may represent a convergent mechanism linking gut dysbiosis to neurodegeneration. Consistent with this, increased circulating H_2_S levels have been reported in patients with AD and dementia [221]. Several of the H_2_S-producing taxa also metabolize dietary choline and carnitine into trimethylamine (TMA), which is subsequently converted in the liver to trimethylamine N-oxide (TMAO) by host flavin monooxygenases [222]. TMAO is a diffusible molecule that readily crosses the BBB and is broadly detected across the CNS [223,224]. Elevated levels of TMAO correlate with cognitive decline, dementia, AD and other PCDs [225–228]. TMAO has been linked to inflammation, mitochondrial dysfunction, UPR regulation and oxidative stress, although its direct effects on proteostasis remain elusive [229,230].

In addition to these sulphur- and nitrogen-associated metabolites, other microbially derived small molecules further expand the spectrum of proteostasis modulators. Another notable species is *S. mutans*, an oral pathogen detected in the brains of AD patients and enriched in the gut microbiota of PD patients [183]. In experimental models, asymptomatic gut colonization by *S. mutans* elevates plasma and brain levels of imidazole propionate, a microbial metabolite that induces PD-like phenotypes [92], further linking microbiota-derived metabolites to systemic proteostasis disruption. Together, these examples illustrate that chemically diverse microbiota-derived metabolites, including SCFAs, bile acids, polyamines, sulphur metabolites and nitrogen-containing compounds, converge on common host pathways regulating protein folding, degradation and cellular stress responses. Collectively, these findings suggest that microbiota-derived metabolites may function as systemic regulators of host proteostasis, with their net effect determined by concentration, localization and host buffering capacity.

## 6. Conclusion and future perspectives

Although the exact aetiology of PCDs remains largely unknown, a growing body of research supports microbial disruption of host proteostasis as a pivotal contributor to the development of these cognitive ailments. The microbiota is no longer recognized as an inert bystander, but instead as a dynamic contributor to overall health, capable of modulating the stability of the host proteome. Functional amyloids, proteolytic enzymes, toxins and other microbial metabolites produced by various bacterial taxa interact with host proteostasis networks, decreasing the cellular ability to buffer proteins in their folded state. However, despite compelling associations between microbiota disruption and PCDs, the interface between microbiology and neurological disorders remains nascent. Much of the available evidence remains correlative or derived from *in vitro* and animal models, and the precise mechanisms through which microbial factors engage with the systemic proteostasis network remain insufficiently characterized.

Advancing this field of research will require mechanistic studies to elucidate how individual microbes and their products interact with the host proteostasis network. Determining the direct molecular interplay between the host proteome and its resident microbes, such as the binding of bacterial amyloids to α-synuclein aggregates or the delivery of microbial proteases into neuronal tissues via EVs, is crucial for distinguishing causality from correlation. Conducting such comprehensive multisystem studies of the microbiota–proteostasis axis will require interdisciplinary collaborations across broad fields, moving away from the traditional neuroscience-centric view.

Studying PCDs within the framework of the microbiota–proteostasis axis presents both significant challenges and transformative opportunities. One major challenge for this research is linking interactions among complex microbial communities to host physiological processes through systems-level integration. However, the prospect of developing innovative diagnostic techniques and therapeutic approaches through microbial biomarker research, dietary interventions, phage-based therapies, precision prebiotics, probiotics, and postbiotics and faecal microbiota transplantation (FMT) marks a paradigm shift in the future of treating PCDs. Numerous microbiome-targeted strategies, including dietary interventions [231,232], FMT [233], prebiotics, probiotics and postbiotics [234,235], have shown promise in experimental and clinical settings. Importantly, however, microbiome-targeted interventions have also produced inconsistent outcomes across studies and patient populations, probably reflecting substantial inter-individual variability in microbiome composition, host genetics, immune responses, diet, medication exposure and disease stage [236,237]. In many cases, microbial engraftment and functional restoration of proteostasis resilience may be transient or incomplete, particularly during advanced stages of disease (figure 1). Nonetheless, while previous efforts to treat neurodegenerative disorders have shown limited clinical success and remained primarily focused on managing symptoms of otherwise terminal illnesses, future strategies may shift to preserving host proteostasis across the lifespan through targeted modulation of the microbiota. Given the recognized importance of microbiota in maintaining host health, it is likely that the infectious disease field may also begin shifting towards the development of microbiome-sparing precision antimicrobials that selectively target bacterial pathogens without contributing to dysbiosis. Collectively, the microbiota–proteostasis axis supports a holistic systems-level framework in which preservation of proteostasis resilience through targeted microbiome modulation may become an important component of future personalized therapeutic strategies for PCDs.

## Data Availability

This article has no additional data.

## Theme

One contribution of 20 to a Theo Murphy meeting issue ‘ProteostaSys: a systems view of proteostasis’.
